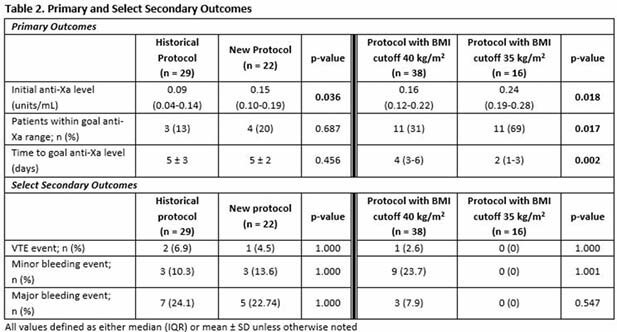# 742 Evaluation of Anti-Xa Levels and Venous Thromboembolism Prophylaxis with Enoxaparin in Burn Patients

**DOI:** 10.1093/jbcr/irad045.217

**Published:** 2023-05-15

**Authors:** Allison Boyd, Todd Walroth, Brett Hartman, Matthew Bjornstad, Alyssa Garelli, Leigh Spera, Cortni Grooms

**Affiliations:** Eskenazi Health, Indianapolis, Indiana; Eskenazi Health, Indianapolis, Indiana; Indiana university/Eskenazi Health, Indianapolis, Indiana; Indiana University Health - Arnett Hospital, Whitestown, Indiana; Butler University, Indianapolis, Indiana; Indiana University School of Medicine/Eskenazi Health, Indianapolis, Indiana; Eskenazi Health, Indianapolis, Indiana; Eskenazi Health, Indianapolis, Indiana; Eskenazi Health, Indianapolis, Indiana; Indiana university/Eskenazi Health, Indianapolis, Indiana; Indiana University Health - Arnett Hospital, Whitestown, Indiana; Butler University, Indianapolis, Indiana; Indiana University School of Medicine/Eskenazi Health, Indianapolis, Indiana; Eskenazi Health, Indianapolis, Indiana; Eskenazi Health, Indianapolis, Indiana; Eskenazi Health, Indianapolis, Indiana; Indiana university/Eskenazi Health, Indianapolis, Indiana; Indiana University Health - Arnett Hospital, Whitestown, Indiana; Butler University, Indianapolis, Indiana; Indiana University School of Medicine/Eskenazi Health, Indianapolis, Indiana; Eskenazi Health, Indianapolis, Indiana; Eskenazi Health, Indianapolis, Indiana; Eskenazi Health, Indianapolis, Indiana; Indiana university/Eskenazi Health, Indianapolis, Indiana; Indiana University Health - Arnett Hospital, Whitestown, Indiana; Butler University, Indianapolis, Indiana; Indiana University School of Medicine/Eskenazi Health, Indianapolis, Indiana; Eskenazi Health, Indianapolis, Indiana; Eskenazi Health, Indianapolis, Indiana; Eskenazi Health, Indianapolis, Indiana; Indiana university/Eskenazi Health, Indianapolis, Indiana; Indiana University Health - Arnett Hospital, Whitestown, Indiana; Butler University, Indianapolis, Indiana; Indiana University School of Medicine/Eskenazi Health, Indianapolis, Indiana; Eskenazi Health, Indianapolis, Indiana; Eskenazi Health, Indianapolis, Indiana; Eskenazi Health, Indianapolis, Indiana; Indiana university/Eskenazi Health, Indianapolis, Indiana; Indiana University Health - Arnett Hospital, Whitestown, Indiana; Butler University, Indianapolis, Indiana; Indiana University School of Medicine/Eskenazi Health, Indianapolis, Indiana; Eskenazi Health, Indianapolis, Indiana; Eskenazi Health, Indianapolis, Indiana; Eskenazi Health, Indianapolis, Indiana; Indiana university/Eskenazi Health, Indianapolis, Indiana; Indiana University Health - Arnett Hospital, Whitestown, Indiana; Butler University, Indianapolis, Indiana; Indiana University School of Medicine/Eskenazi Health, Indianapolis, Indiana; Eskenazi Health, Indianapolis, Indiana

## Abstract

**Introduction:**

Previous studies have found that standard dose enoxaparin is inadequate in achieving goal prophylactic anti-Xa levels in burn patients. A new venous thromboembolism (VTE) prophylaxis protocol was implemented at our institution (Table 1). The objective of this study was to assess the efficacy and safety of the updated VTE prophylaxis protocol to determine if goal anti-Xa levels can be achieved earlier compared to the previous enoxaparin dosing protocol.

**Methods:**

This was a retrospective cohort study evaluating a historic protocol group from 3/1/21 to 5/31/21 and a new protocol group from 10/1/21 to 12/31/21. Revisions were made to the new protocol group and evaluated from 7/1/22 to 8/31/22. Adult patients admitted with cutaneous and/or inhalation burn who received enoxaparin for VTE prophylaxis and had anti-Xa monitoring were included. The primary endpoint was protocol efficacy defined as percentage of initial anti-Xa levels within goal range and time to goal anti-Xa level. Secondary endpoints included protocol adherence, rate of VTE occurrence, number of missed or held doses if VTE occurred, correlation to published enoxaparin dosing equations in burn patients, and bleeding events.

**Results:**

Results can be found in Table 2. The median (IQR) initial anti-Xa level in the historical (n=29) vs. new protocol group (n=22) was 0.09 (0.04-0.14) units/mL vs. 0.15 (0.10-0.19) units/mL (p=0.036). After adjusting the BMI cutoff in the new protocol from 40 kg/m^2^ to 35 kg/m^2^, the median initial anti-Xa was 0.16 (0.12-0.22) units/mL vs. 0.24 (0.19-0.28) units/mL (p=0.018). The mean ± SD time for patients to achieve goal anti-Xa level was 5 ± 3 days in the historical protocol and 5 ± 2 days in the new protocol group (p = 0.456). After protocol modification, the median time to goal anti-Xa level was 4 (3-6) days vs. 2 (1-3) days (p=0.002). No differences were noted in secondary outcomes.

**Conclusions:**

The new protocol resulted in a higher initial anti-Xa level with more patients within goal anti-Xa range which was reached in less time after modification of the BMI cutoff for weight-based dosing. The maximum anti-Xa level in the modified group was still within the prophylactic range with the new protocol, indicating no increase in supratherapeutic levels compared to previously utilized dosing strategies and there were no differences in major or minor bleeding events which supports its safety.

**Applicability of Research to Practice:**

This protocol can be replicated and utilized by other burn centers with the potential for a multicenter analysis and provides a framework for developing standardized VTE prophylaxis dosing recommendations with enoxaparin in burn patients.